# Minimally Invasive Surgery With Sentinel Lymph Node Mapping in Elderly Patients With Early-Stage Endometrial Cancer: A Subgroup Analysis of a Prospective Cohort Study

**DOI:** 10.7759/cureus.87200

**Published:** 2025-07-02

**Authors:** Shinichi Togami, Nozomi Furuzono, Hitomi Tsuruzono, Mika Fukuda, Hiroaki Kobayashi

**Affiliations:** 1 Department of Obstetrics and Gynecology, Faculty of Medicine, Kagoshima University, Kagoshima, JPN

**Keywords:** age, endometrial cancer, minimally invasive surgery, prognosis, sentinel lymph node

## Abstract

Objective

The objective of this study is to evaluate the surgical safety and oncological outcomes of minimally invasive surgery (MIS) with sentinel lymph node (SN) mapping in older adults (≥70 years) with early-stage endometrial cancer, as part of a prospective cohort study. * *

Methods

This study is a subgroup analysis of a prospective cohort comprising 204 patients with International Federation of Gynecology and Obstetrics stage IA endometrial cancer who underwent MIS with SN mapping at a single tertiary center between December 2016 and April 2022. Patients were categorized into two groups based on age: <70 years and ≥70 years. Perioperative outcomes, SN detection rates, and survival outcomes were compared between the two groups.

Results

Of the total cohort, 29 patients were aged ≥70 years. Non-endometrioid histology (6.9% vs. 1.1%, P = 0.039) and deep myometrial invasion (27.6% vs. 11.4%, P = 0.019) were more common in the older adults group. Although bilateral SN detection was lower in elderly patients (72.4% vs. 91.4%, P = 0.011), no intraoperative complications occurred in this group. Postoperative complication rates and recurrence-free survival (93% vs. 97.1%, P = 0.29) were comparable across groups.

Conclusion

MIS with SN mapping is a safe and feasible approach for older adults with early-stage endometrial cancer. Despite higher-risk pathological features, older patients had equivalent surgical and oncological outcomes. Age alone should not preclude surgical treatment; instead, decisions should be based on functional status and preoperative evaluation.

## Introduction

Endometrial cancer (EC) is the most prevalent gynecologic malignancy in developed nations, and its incidence continues to rise, driven in part by increasing life expectancy, obesity, and metabolic syndrome. The median age at diagnosis is approximately 68 years, and more than 40% of cases occur in women aged 65 or older [1]. With a globally aging population, the number of elderly patients with EC is expected to grow substantially, raising critical questions about optimal surgical management in this demographic.

Surgical intervention remains the cornerstone of treatment for early-stage EC. Standard procedures include total hysterectomy with bilateral salpingo-oophorectomy, along with nodal assessment using either pelvic and para-aortic lymphadenectomy or sentinel lymph node (SN) mapping. In older adults, however, the extent of surgery is often limited due to concerns over increased perioperative morbidity, prolonged recovery, and comorbid conditions. These concerns have contributed to a tendency toward undertreatment, including omission of lymph node assessment, even in patients who would otherwise be appropriate candidates [1,2].

Minimally invasive surgery (MIS), including laparoscopic and robotic approaches, has dramatically transformed the management of gynecologic malignancies over the past two decades. Compared to the post-operation period of open surgery, MIS is associated with reduced blood loss, fewer wound complications, shorter hospitalization, and faster return to daily activities. Importantly, these benefits are particularly relevant for older adults, who may be more susceptible to complications and functional decline following major surgeries [1,2].

Large-scale database studies have demonstrated the feasibility and potential survival benefits of MIS in elderly women with EC. For instance, analyses from the U.S. National Cancer Database showed that patients aged ≥65 years who underwent MIS had improved five-year overall survival (OS) compared to those who received open surgery. These findings suggest that MIS not only reduces short-term morbidity but may also positively impact long-term outcomes in elderly populations [1].

Despite this, real-world practice still shows disparities in the use of MIS for older patients. Several studies have noted that older women with EC are less likely to undergo MIS and lymph node staging than younger patients. Luzarraga-Aznar et al. reported that only 58.2% of patients aged ≥75 years received MIS, compared to 74.8% in younger patients [2]. Moreover, pelvic and para-aortic lymphadenectomy was performed significantly less often in older patients, leading to a higher rate of surgical understating (37% in ≥75 years vs. 15.2% in <75 years, p = 0.002) [2].

These discrepancies may be partially explained by concerns about the risks of prolonged surgery, anesthetic complications, or technical difficulties in the setting of previous surgeries and adhesions. However, multiple studies have shown that older patients do not experience significantly higher rates of intraoperative or postoperative complications with MIS compared to younger patients. Furthermore, when SN biopsy is used instead of full lymphadenectomy, the surgical burden is further reduced without compromising diagnostic accuracy.

SN biopsy has emerged as a less invasive and increasingly accepted alternative to systematic lymphadenectomy for staging in EC. When performed correctly using tracers such as indocyanine green (ICG) or radiocolloid, SN mapping offers high detection rates, low false-negative rates, and substantially reduced morbidity [3-6]. Multiple guidelines have endorsed it as an appropriate method for lymph node assessment in clinically early-stage EC [7]. The advantages of SN mapping are especially pronounced in elderly patients, where the goal is to maintain oncological accuracy while minimizing operative risk.

However, as with MIS, the adoption of SN mapping in older populations has been inconsistent. Technical concerns, such as altered lymphatic flow or decreased tracer uptake, may make some surgeons hesitant to perform SN mapping in older individuals. Furthermore, the evidence base specific to older patients undergoing MIS with SN mapping remains limited, and few studies have comprehensively assessed outcomes in this subgroup.

In this context, our study was designed to evaluate the perioperative and oncological outcomes of older adults with early-stage EC who underwent MIS with SN mapping. We conducted a cohort analysis of 204 patients who underwent surgery using this combined minimally invasive approach. Patients were stratified by age, focusing on comparing outcomes between those aged ≥70 and those <70 years.

We primarily aimed to assess whether age affects the safety and feasibility of MIS with SN mapping. We examined intraoperative and postoperative complication rates, conversion to laparotomy, operative time, blood loss, and length of hospital stay.

## Materials and methods

This study was conducted as a subgroup analysis of a prospective observational study approved by the Institutional Review Board (IRB) of Kagoshima University Hospital (IRB No. 20-K04). All patients provided written informed consent prior to enrollment. The study complied with the Declaration of Helsinki and relevant institutional ethical guidelines. The cases included in this study were treated between April 2017 and March 2022.

Population (P)

Eligible participants included patients who were preoperatively diagnosed with endometrioid endometrial carcinoma via endometrial biopsy and clinically staged as International Federation of Gynecology and Obstetrics (FIGO 2008) stage IA based on imaging studies, including contrast-enhanced computed tomography (CT) and magnetic resonance imaging (MRI) [8]. All patients underwent MIS, including total hysterectomy, bilateral salpingo-oophorectomy, and SN mapping. Patients with radiological evidence of pelvic or para-aortic lymph node enlargement were excluded. Clinical, pathological, and surgical data were retrospectively extracted from electronic medical records.

Comorbidities, including cardiovascular disease, diabetes mellitus, and respiratory disorders, were reviewed and recorded. These conditions were not exclusion criteria but were considered during the preoperative anesthesia risk assessment. All patients were evaluated preoperatively by the gynecologic oncology and anesthesiology teams to determine surgical eligibility and perioperative risk. Preoperative imaging and endometrial biopsy were used to confirm the clinical stage and histologic subtype.

Intervention (I)

All patients underwent surgery via a minimally invasive approach, either laparoscopic or robot-assisted. Patients were assigned to surgical modality sequentially based on operating room availability, without selection bias. Robotic procedures were performed using either the da Vinci Xi Surgical System (Intuitive Surgical Inc., Sunnyvale, CA) or the hinotori™ Surgical Robot System (Medicaroid Corporation, Kobe, Japan). Uterine manipulators were not used in any procedure. To prevent tumor spillage, the external cervical os was closed with absorbable sutures prior to surgery initiation.

Comparison (C)

The study population was divided into two groups according to age at the time of surgery: <70 years and ≥70 years. Comparative analyses were performed between these two age groups to assess differences in surgical approach, perioperative outcomes, SN detection rates, and complication rates.

Outcome (O)

The primary outcome was perioperative morbidity, assessed according to the Common Terminology Criteria for Adverse Events. Secondary outcomes included operative time, estimated blood loss, length of hospital stay, SN detection rate, and the rate of surgical staging adequacy.

Sentinel lymph node mapping procedure

SN mapping was performed using a hybrid technique combining radiocolloid and fluorescence imaging. On the day prior to surgery, 111 MBq of technetium-99m-labeled phytate colloid was injected into the cervix at four quadrants. Planar lymphoscintigraphy and single-photon emission CT with CT fusion (SPECT-CT) were used to preoperatively assess the anatomical location of SN. Immediately before surgery, 1 mL of 10-fold diluted ICG was injected into the cervix at the 3 and 9 o’clock positions. Intraoperatively, SNs were visualized using a near-infrared fluorescence imaging system integrated into the laparoscopic or robotic platforms. In cases with unilateral or no SN detection, a systemic pelvic lymphadenectomy was performed to ensure accurate assessment of nodal status.

Statistical analysis

Statistical analyses were performed using JMP software (SAS Institute Inc., Cary, NC). Continuous variables were expressed as medians with ranges and compared using the Wilcoxon rank-sum test. Categorical variables were compared using the chi-square test or Fisher’s exact test, as appropriate. A two-tailed P-value of <0.05 was considered statistically significant.

## Results

Patient characteristics and surgical outcomes

A total of 204 patients with FIGO stage IA EC who underwent MIS with SN mapping between December 2016 and April 2022 at Kagoshima University Hospital were included in the analysis. Of these, 175 (85.8%) were aged <70 years, and 29 (14.2%) were aged ≥70 years. Detailed patient characteristics are presented in Table 1.

**Table 1:** Clinicopathological characteristics of the 204 patients included in this study Data are given as median (range) or n (%). Statistical significance of differences between groups is tested by the χ² test for categorical data and by the Wilcoxon signed-rank test for continuous parameters. P-values < 0.05 were considered statistically significant and are marked with “*.” BMI: body mass index, FIGO: International Federation of Gynecology and Obstetrics, LVSI: lympho-vascular space invasion

Characteristic	Age < 70 (n = 175)	Age ≥ 70 (n = 29)	Test Statistic	P-value
Median BMI (kg/m^2^)	26.5 (16.4-53.1)	22.5 (19-32.7)	Z = -2.55	0.011*
Final pathology			χ² = 4.28	0.039*
Endometrioid	173 (98.9%)	27 (93.1%)	-	-
Non-endometrioid	2 (1.1%)	2 (6.9%)	-	-
LVSI			χ² = 2.07	0.15
No	153 (87.4%)	28 (96.6%)	-	-
Yes	22 (12.6%)	1 (3.4%)	-	-
Myometrial invasion			χ² = 5.49	0.019*
<1/2	155 (88.6%)	21 (72.4%)	-	-
≥1/2	20 (11.4%)	8 (27.6%)	-	-
FIGOstage (2009)			χ² = 18.44	0.0004*
IA	156 (89.2%)	20 (69%)	-	-
IB	9 (5.1%)	8 (27.6%)	-	-
II	2 (1.1%)	1 (3.4%)	-	-
IIIC1	8 (4.6%)	0	-	-
Surgical procedure			χ² = 9.29	0.0023*
Robot	123 (70.3%)	12 (41.4%)	-	-
Laparoscopy	52 (29.7%)	17 (58.6%)	-	-
Median operation time, min (range)	199 (99-555)	172 (87-304)	Z = -1.55	0.12
Median blood loss, mL (range)	15 (2-453)	20 (5-185)	Z = -0.23	0.82
Conversion to laparotomy	0	0	-	-
Intra-operation complications			χ² = 0.51	0.48
No	172 (98.3%)	121 (97.6%)	-	-
Yes	29 (100%)	0	-	-
Post-operation complications			χ² = 0.85	0.36
No	170 (97.1%)	29 (100%)	-	-
Yes	5 (2.9%)	0	-	-
Median length of hospital stays, days (range)	6 (4-68)	6 (4-15)	Z = 0.12	0.9
Adjuvant therapy			χ² = 2.05	0.36
No	146 (83.4%)	21 (72.4%)	-	-
Chemotherapy	25 (14.3%)	7 (24.1%)	-	-
Radiotherapy	4 (2.3%)	1 (3.5%)	-	-
Recurrence			χ² = 0.03	0.86
No	170 (97.1%)	28 (96.6%)	-	-
Yes	5 (2.9%)	1 (3.4%)	-	-

Patients aged ≥70 years had a significantly lower body mass index than those aged <70 (median 22.5 kg/m²; range 17.8-27.4 vs. 26.5 kg/m²; range 16.9-43.4; P = 0.011). The proportion of patients with non-endometrioid histology was higher in the ≥70 years group (2/29, 6.9%) compared to the <70 group (2/175, 1.1%) (P = 0.039). Regarding histological subtypes, among patients aged <70 years, two had serous carcinoma and one had mixed carcinoma. In the ≥70 years group, one patient had serous carcinoma and one had clear cell carcinoma. Deep myometrial invasion (>50%) was observed more frequently in patients ≥70 years (8/29, 27.6%) than in those <70 years (20/175, 11.4%) (P = 0.019). Similarly, stage IB disease was more prevalent in the older group (8/29, 27.6%) compared to the younger group (9/175, 5.1%) (P = 0.0004).

Regarding surgical modality, robotic surgery was performed in 123/175 patients (70.3%) aged <70 years and 12/29 patients (41.4%) aged ≥70 years, while laparoscopic surgery was performed in 52/175 patients (29.7%) and 17/29 patients (58.6%), respectively (P = 0.0023). No conversions to laparotomy occurred in either group.

Operative time was slightly longer in patients aged <70 years (median 199 min; range 110-417 min) than in those ≥70 (172 min; range 120-319 min), although the difference was not statistically significant (P = 0.12). Estimated blood loss was comparable between the two groups (15 mL; range 0-300 vs. 20 mL; range 0-200, P = 0.82). Median length of hospital stay was six days in both groups (range 4-18 vs. 4-15, P = 0.90).

Intraoperative complications occurred in 3/175 patients (1.7%) in the <70 years group and in no patient in the ≥70 years group (0/29, 0%) (P = 0.48). The intraoperative complications included one case each of bladder, small bowel, and rectal injuries. All cases were promptly recognized intraoperatively and managed without requiring conversion to laparotomy.

Postoperative complications were observed in 5/175 patients (2.9%) in the <70 years group and no patient in the ≥70 years group (0/29, 0%) (P = 0.36). The postoperative complications consisted of two cases of ileus, two cases of pelvic abscess, and one case of ureteral stricture. All postoperative events were managed conservatively, and no patient required reoperation or invasive intervention. Adjuvant therapy was administered to 19/175 patients (10.9%) in the <70 years group and 5/29 patients (17.2%) in the ≥70 years group (P = 0.36).

Sentinel lymph node mapping outcomes

SN mapping results are presented in Table 2. The rate of bilateral SN detection was significantly lower in the ≥70 years group than in the <70 years group (72.4% vs. 91.4%, P = 0.011), while unilateral detection and mapping failure occurred more frequently in the elderly group (17.3% and 10.3%, respectively, vs. 5.7% and 2.9%). However, the median number of sentinel nodes removed was comparable between the two groups (two nodes; range 1-5, P = 0.17). No patient in either group developed pelvic lymphocele. Lower extremity lymphedema was observed in two patients in the <70 years group (1.1%) and in none of the ≥70 years patients (P = 0.56).

**Table 2:** Sentinel lymph node-related outcomes of the 204 patients included in this study Statistical significance of differences between groups was tested by the χ² test for categorical data and by the Wilcoxon signed-rank test for continuous parameters. P-values < 0.05 were considered statistically significant and are marked with “*.” SN, sentinel lymph node

Characteristic	Age < 70 (n = 175)	Age ≥ 70 (n = 29)	Test Statistic	P-value
SN mapping			χ² = 9.07	0.011*
Bilateral	160 (91.4%)	21 (72.4%)	-	-
Unilateral	10 (5.7%)	5 (17.3%)	-	-
None	5 (2.9%)	3 (10.3%)	-	-
Median number of SN removed (range)	2 (1-5)	2 (1-5)	Z = -1.38	0.17
Lower extremity lymphedema			χ² = 0.34	0.56
No	173 (98.9%)	29 (100%)	-	-
Yes	2 (1.1%)	0	-	-
Pelvic lymphocele			-	-
No	175 (100%)	29 (100%)	-	-
Yes	0	0	-	-

Oncologic outcomes

Recurrence occurred in 5/175 patients (2.9%) aged <70 and in 1/29 patients (3.4%) aged ≥70 (P = 0.86). Among the six recurrence cases, three were isolated vaginal cuff recurrences, and the remaining three involved peritoneal dissemination. All six patients who experienced recurrence had endometrioid histology.

**Figure 1:**
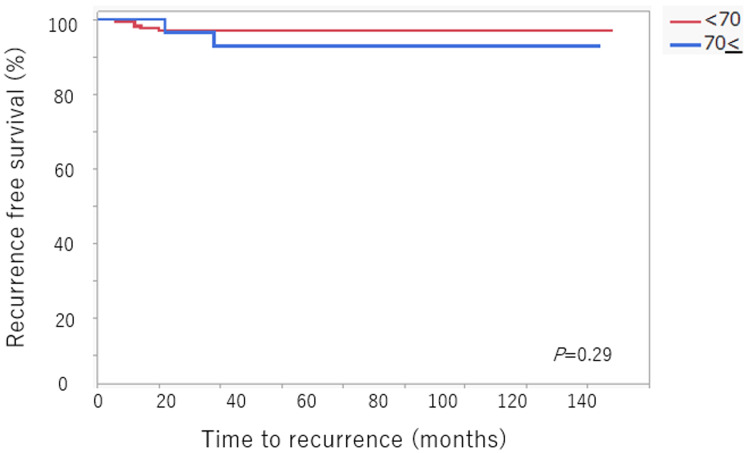
Five-year recurrence-free survival rate

**Figure 2:**
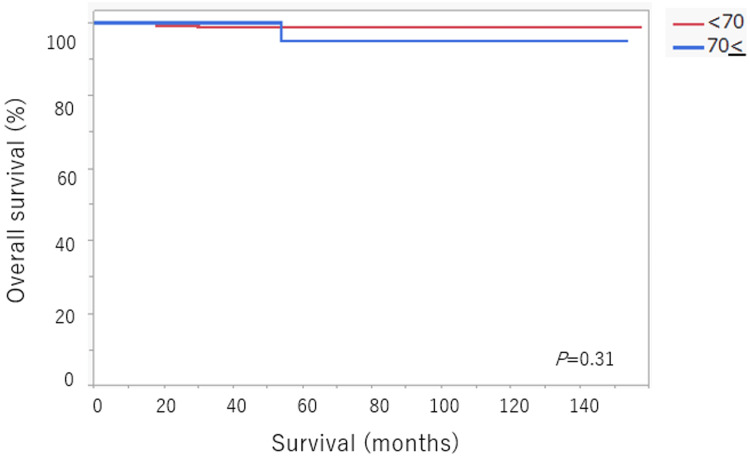
Five-year overall survival rate

The median follow-up duration was 54 months (range 8-84). Kaplan-Meier survival curves are shown in Figure 1 and Figure 2.

Recurrence-free survival (RFS) was similar between groups. The five-year RFS rate was 97.1% in the <70 years group and 93% in the ≥70 years group, with no statistically significant difference (P = 0.29) (Figure 1).

Similarly, OS did not differ significantly between age groups. The five-year OS rate was 98.8% in the <70 years group and 95% in the ≥70 years group (P = 0.31) (Figure 2). These findings suggest that age ≥70 years was not associated with inferior oncologic outcomes in patients undergoing MIS with SN mapping for early-stage EC.

## Discussion

In the present study, 204 patients with FIGO stage IA EC underwent MIS with SN mapping. Of these, 29 were aged ≥70 years. Despite a higher incidence of adverse histologic features, surgical outcomes were comparable. No intraoperative complications occurred in the ≥70 years group, and severe postoperative complications were absent in both. These findings are consistent with earlier reports demonstrating the safety and feasibility of MIS in older patients [9-11].

The broader literature supports the notion that age alone should not preclude the use of MIS. For example, Bourgin et al. reported that older patients (≥75 years) experienced similar complication rates compared to younger patients, and although MIS was performed less frequently in the older group, it was not associated with increased morbidity [12]. Similarly, Gallotta et al. found the feasibility, safety, and good short-term outcomes of robotic surgery in elderly and very elderly patients with gynecologic cancer [13]. These findings reinforce the view that MIS should be equally offered to elderly patients who are appropriate surgical candidates.

An important component of modern EC surgery is the incorporation of SN mapping, which has replaced systematic lymphadenectomy in many institutions due to its lower associated morbidity and high diagnostic accuracy [3,4]. The utility of SN mapping is particularly significant in older patients, as it allows for accurate staging with minimal invasiveness. In this study, although the bilateral SN detection rate was lower in the ≥70 years group (72.4%) compared to the <70 years group (91.4%), the total number of SNs retrieved did not differ significantly. No patient in either group experienced complications related to lymph node dissection, such as lymphocele or lymphedema, suggesting that SN mapping is not only feasible but also safe in the elderly population.

Despite the growing body of evidence supporting MIS and SN mapping, underutilization persists among older women. Bourgin et al. demonstrated that patients aged ≥75 years were less likely to undergo SN mapping and were more often surgically understaged compared to their younger counterparts [2]. This practice may be driven by unfounded concerns regarding the reliability of lymphatic mapping in older patients, particularly in the context of age-related changes in lymphatic flow or comorbidities that may interfere with tracer uptake. However, the results of our study and others challenge this assumption, emphasizing that SN mapping can and should be implemented in older adults to avoid unnecessary omission of nodal staging and improve oncologic risk stratification.

In terms of oncologic outcomes, this study observed no statistically significant differences in RFS or OS between the age groups. The recurrence rate in the ≥70 years group was 3.4%, comparable to the 2.9% observed in the <70 years group. In the present study, six patients (2.9%) experienced recurrence despite being classified as FIGO stage IA. This recurrence rate is consistent with previous reports. For example, Nwachukwu et al. evaluated recurrence in 222 patients with stage IA EC and observed 17 recurrences (7.65%) [14]. Therefore, the recurrence rate observed in our cohort does not indicate a substantially higher risk and remains within the expected range. All recurrences were localized either at the vaginal cuff or in the peritoneal cavity and were managed effectively with standard adjuvant treatments. These findings are supported by previous investigations, which showed no difference in survival outcomes between elderly and younger patients following MIS for EC [1,13,15]. Furthermore, robotic surgery, which may offer ergonomic benefits and enhanced precision, has been shown in several studies to reduce estimated blood loss and maintain low complication rates even in older populations [13,15].

However, it is important to acknowledge the physiological challenges specific to robotic surgery in older adults. Robotic-assisted procedures generally require a steeper Trendelenburg position than conventional laparoscopy, which can increase cardiopulmonary strain due to elevated intra-abdominal pressure and cranial displacement of the diaphragm [15,16]. These physiological changes may impair respiratory mechanics and venous return, particularly in frail or comorbid elderly patients. Therefore, while robotic platforms offer surgical advantages such as improved dexterity, enhanced visualization, and reduced surgeon fatigue, their use must be balanced with the need for vigilant anesthetic management and comprehensive preoperative evaluation in older adults with limited cardiopulmonary reserve.

The present study also demonstrated that older patients were more likely to present with adverse pathological features such as non-endometrioid histology and deep myometrial invasion, consistent with previous reports suggesting that advanced age is associated with higher-risk disease [10,11]. Nevertheless, these factors did not translate into worse outcomes, potentially due to effective surgical staging and appropriate adjuvant therapy. This underscores the importance of thorough staging in older patients, even when comorbidities are present, as an omission of nodal assessment can lead to undertreatment and compromise survival.

Although the results of this study are encouraging, some limitations must be acknowledged. First, the number of patients aged ≥70 years was relatively small (n = 29), which reduces the statistical power to detect rare outcomes such as disease recurrence or mortality. Additionally, frailty was not formally assessed using validated geriatric indices such as the modified frailty index or the G8 screening tool, both of which have been shown to more accurately predict perioperative risks than chronological age alone [17]. Incorporating frailty assessments in future studies could help optimize patient selection and perioperative management strategies. Moreover, although the study enrolled patients up to 2022, molecular classification or genomic profiling was not performed. Future investigations, including molecular features, may further clarify recurrence risk and guide individualized treatment strategies.

## Conclusions

In conclusion, our study suggests that MIS with SN mapping may be a safe and effective option for patients aged 70 years or older with early-stage EC. Despite a higher frequency of adverse histologic features and a slightly lower SN detection rate, elderly patients demonstrated comparable surgical safety and oncologic outcomes to younger patients. Chronological age alone should not preclude consideration of standard surgical treatment. Rather, decisions should be guided by functional status, oncologic risk, and comprehensive preoperative assessment, including frailty evaluation. Larger prospective multicenter studies incorporating geriatric assessment tools are warranted to validate these findings and support personalized surgical decision-making in this growing population.
